# Pulmonary perfusion and NYHA classification improve after cardiac resynchronization therapy

**DOI:** 10.1007/s12350-021-02848-8

**Published:** 2021-11-08

**Authors:** Mariam Al-Mashat, Rasmus Borgquist, Marcus Carlsson, Håkan Arheden, Jonas Jögi

**Affiliations:** 1 Clinical Physiology, Department of Clinical Sciences Lund, Skåne University Hospital, Lund University, Entrégatan 7, 22185 Lund, Sweden; 2 Cardiology, Arrhythmia Section, Department of Clinical Sciences Lund, Skåne University Hospital, Lund University, Lund, Sweden

**Keywords:** Cardiac resynchronization therapy, echocardiography, heart failure, NYHA classification, ventilation/perfusion single-photon emission computed tomography

## Abstract

**Background:**

Evaluation of cardiac resynchronization therapy (CRT) often includes New York Heart Association (NYHA) classification, and echocardiography. However, these measures have limitations. Perfusion gradients from ventilation/perfusion single-photon emission computed tomography (V/P SPECT) are related to left-heart filling pressures and have been validated against invasive right-heart catheterization. The aim was to assess if changes in perfusion gradients are associated with improvements in heart failure (HF) symptoms after CRT, and if they correlate with currently used diagnostic methods in the follow-up of patients with HF after receiving CRT.

**Methods and results:**

Nineteen patients underwent V/P SPECT, echocardiography, NYHA classification, and the quality-of-life scoring system “Minnesota living with HF” (MLWHF), before and after CRT. CRT caused improvement in perfusion gradients from V/P SPECT which were associated with improvements in NYHA classification (*P* = .0456), whereas improvements in end-systolic volume (LVESV) from echocardiography were not. After receiving CRT, the proportion of patients who improved was lower using LVESV (*n* = 7/19, 37%) than perfusion gradients (*n* = 13/19, 68%). Neither change in perfusion gradients nor LVESV was associated with changes in MLWHF (*P* = 1.0, respectively).

**Conclusions:**

Measurement of perfusion gradients from V/P SPECT is a promising quantitative user-independent surrogate measure of left-sided filling pressure in the assessment of CRT response in patients with HF.

**Supplementary Information:**

The online version contains supplementary material available at 10.1007/s12350-021-02848-8.

## Introduction

Heart failure is a complex syndrome with a mortality that is comparable with or even worse than most malignancies.^1^ Heart failure also affects quality of life and life expectance.^2^

Cardiac resynchronization therapy (CRT) is a treatment option in patients with heart failure with left bundle branch block (LBBB), wide QRS complex, and left-ventricular ejection fraction (LVEF) ≤ 35% that do not show improvement despite optimal pharmacological treatment.^3^ CRT results in decreased morbidity and fewer cardiovascular events compared with pharmacological treatment,^4–6^ as well as improvement in EF and symptoms.^5^ On the other hand, around 25–40% of patients do not derive measurable benefit from CRT, most likely due to a combination of factors including suboptimal lead placement, suboptimal device programming, and presence of myocardial scar.^7^ There is no single clinical finding or test that is able to accurately predict patients’ responses to CRT on its own. The evaluation and follow-up of CRT are multifaceted and usually include functional evaluation with New York Heart Association (NYHA) classification, a 6-minute walk test, N-terminal pro-B type natriuretic peptide, and electrocardiogram (ECG). Echocardiography is the most widely used imaging method for assessment of positive response after CRT, which is especially shown by a decrease in left-ventricular end-systolic volume (LVESV) of more than 15%.^8^ A decrease in LVESV is correlated to an improved long-term prognosis after CRT treatment. Echocardiography and the other currently used methods are to a certain degree observer dependent and subjective.^9–11^ Pulmonary artery wedge pressure (PAWP) from invasive right-heart catheterization has also been used in the follow-up of CRT patients, and it has been shown that PAWP improves after CRT.^12^ PAWP is objective and is used to estimate the degree of pulmonary congestion in patients with heart failure. Even though PAWP is regarded as the reference to assess pulmonary congestion, its role is limited by its invasiveness, and alternative objective non-invasive imaging methods that can quantify the effect of CRT are warranted. It has been shown that perfusion gradients derived automatically from ventilation/perfusion single-photon emission computed tomography (V/P SPECT) can be used as a surrogate for PAWP in the diagnosis of pulmonary congestion in heart failure,^13^ as well as in the follow-up after heart transplantation.^14^ The value of V/P SPECT in the non-invasive evaluation of CRT response, however, is unknown. The aim of this study was, therefore, to assess if (a) changes in perfusion gradients from V/P SPECT are associated with improvements in heart failure symptoms after CRT and (b) perfusion gradients from V/P SPECT correlate with currently used diagnostic methods in the follow-up of patients with heart failure after receiving CRT.

## Material and Methods

### Study Design

Twenty-two patients with heart failure (73 [66-77] years, 5 women), who were candidates for receiving CRT, were prospectively included between 2013 and 2017 at Skåne University Hospital, Lund, Sweden. The patients were examined with V/P SPECT and echocardiography before, and six months after, CRT. The patients had optimal pharmacological treatment that was considered appropriate both before and after CRT. The current study is a sub-study of a larger clinical trial study (ClinicalTrials.gov Identifier: NCT01426321, CRT clinic) where 102 patients were included. The inclusion criteria were wide QRS complex on ECG > 120 milliseconds, symptomatic heart failure (NYHA-class II-IV) despite optimal treatment with medications, EF ≤ 35% and accepted for CRT-P or CRT-D. For the V/P SPECT examination, patients older than 40 years were considered. Exclusion criteria were chronic atrial fibrillation, life expectancy below 12 months, pregnancy, myocardial infarction less than 3 months before enrollment, severe kidney failure with glomerular filtration rate < 30 mL/min, significant valve disease, and patients who were incapable of giving written informed consent.

The ethical review board at Lund University approved the study, and informed consent was obtained from all patients before inclusion.

### CRT

The CRT was performed, after randomization, either according to clinical routine or after extended diagnostic examinations (intervention group) using echocardiography, computed tomography, and magnetic resonance imaging. The operator could take part of the results from these examinations and was, therefore, guided to find optimal placement of the CRT electrode. The main results and methodology of the study have been published previously.^15^

For this sub-study, twenty-two CRT patients from the main study were included and were complemented with a V/P SPECT examination. Eight of the 22 patients had been randomized to extended diagnostic examinations before CRT in the main study, thirteen patients had been randomized to standard diagnostic evaluation and served as controls, and one patient was not randomized because of normal EF at baseline. Four patients in the control group and three patients in the intervention group were LVESV (from echocardiography) responders. The results of the primary study were negative, and therefore, the patients are treated as one group for this sub-study.

### Image Acquisition

#### V/P SPECT

The V/P SPECT examination was performed according to the European guidelines, as a one-day protocol.^16,17^ V/P SPECT enables the assessment of the distribution of ventilation and perfusion in the lungs. The ventilation study was performed before the perfusion study; both were performed in the same supine position. For the assessment of pulmonary ventilation, Technegas (Cyclomedica Ltd, Dublin, Ireland) was inhaled until a total of 25 MBq reached the lungs. The images were then acquired using a dual-head gamma camera (Discovery NM/CT 670 or Discovery NM 630, GE healthcare Sverige AB, Danderyd, Sweden), with an extended low-energy general purpose collimator and an acquisition time of 10 seconds in 120 projections. For the assessment of the pulmonary perfusion, 120 MBq of 99m-Technetium-labeled macroaggregated albumin (TechneScan LyoMAA; Mallinckrodt Medical BV, Petten, Netherlands) was injected intravenously. The acquisition time for the perfusion study was five seconds per projection. Reconstruction of the V/P SPECT images was made with iterative reconstruction, using Ordered Subset Expectation Maximization, and the images were qualitatively assessed using Oasis Pulmogam software (Segami corp., Columbia, MD, USA).

#### Perfusion gradients

Perfusion gradients were automatically derived from the perfusion SPECT images using a previously developed and validated algorithm.^13,18^ In short, the algorithm calculated the total amount of activity of the injected 99m-Technetium-labeled macroaggregated albumin in the total lung volume for both lungs. To avoid partial volume effect and artifacts, one cm of the lung borders and large vessels were excluded. Thereafter, the algorithm created a three-dimensional regression of the perfusion activity in the lungs, where the slope in the sagittal plane represents the perfusion gradients from the posterior to the anterior direction of the lungs. The unit of the perfusion gradients is %-counts/cm and compared with the normalized maximum radio activity, it represents the change in counts per centimeter lung. Patients with negative perfusion gradients (below 0% counts/cm) have normal perfusion pattern in the lungs, while patients with positive perfusion gradients (over 0% counts/cm) have a redistribution of the pulmonary perfusion to anterior parts of the lungs.^13^ The perfusion gradient increases gradually with increasing PAWP.

#### Echocardiography

A Vivid 7 (GE Medical, Horten, Norway) ultrasound system was used for the transthoracic echocardiography examinations. The examinations were performed blinded according to guidelines^19^ by one experienced echocardiographer with more than 10 years of experience. The endocardial contours were manually delineated for the 21/22 patients in the 2- and 4-chamber views, and LV end-diastolic volume (EDV) and LV end-systolic volume (ESV) and EF were then calculated using the biplane Simpson method. In one patient, the volumes were calculated in the 2-chamber view.

### Image Analysis

#### Quantitative assessment of V/P SPECT

The quantitative assessment of the V/P SPECT images was made using the perfusion gradients algorithm. A normal perfusion gradient in a healthy control with predominantly dorsal/posterior perfusion results in a negative value (below 0 %-counts/cm).^18^ In patients with heart failure and elevated filling pressures, there is a ventral/anterior shift in pulmonary perfusion resulting in a positive value (above 0% counts/cm).^13^

#### Echocardiography

The workstation Echopac BT12 (GE Medical, Horten, Norway) was used for the analysis of the echocardiographic images.

### Endpoints

The endpoints that were used in order to find out if the patients were responders or not were LVESV (≥ 15% relative reduction)^8^ and LVEF (≥ 10% absolute increase) from echocardiography. In addition, NYHA classification (improved ≥ 1 class)^20,21^ and a quality-of-life scoring system for heart failure (Minnesota living with heart failure (MLWHF)) (≥ 10% relative reduction)^22^ were used as clinical endpoints.

### Statistical Analysis

GraphPad Prism 7.04 software (GraphPad Software, Inc., La Jolla, CA, USA) and SPSS version 23.0 (IBM, Armonk, NY, USA) were used for statistical analysis.

Histograms were visually assessed and used to test if the data in the study were normally distributed or not. The data were not normally distributed and, therefore, are presented as median and inter-quartile range [IQR]. The non-parametric Wilcoxon-signed ranks test was used to investigate the difference in perfusion gradients and LVESV in patients before and after receiving CRT. Linear correlation was used when testing the perfusion gradients against the LVESV. When investigating the difference between improved/non-improved perfusion gradients vs improvement in NYHA, MLWHF quality-of-life scoring system, LVESV and EF, Fisher’s exact test was used. The same statistical test was used when investigating the difference between improved/non-improved LVESV and NYHA, MLWHF quality-of-life scoring system, LVEF, and perfusion gradients. Kruskal–Willis test was used to test the association between NYHA classification and 6-minute walk test, left-ventricular end-diastolic volume (LVEDV), and LVEF. Results with *P* value < .05 were considered statistically significant.

## Results

Three of the 22 included patients were lost to follow-up because they declined the follow-up study, leaving 19 patients (74 [65-77] years, 5 women) for statistical analysis. Table 1 shows patient characteristics for the 19 patients.

**Table 1:** Patient characteristics on responders and non-responders according to perfusion gradients from V/P SPECT

	Responders according to improved perfusion gradients	Non-responders according to no improvement in perfusion gradients
Total number of patients	13	6
Age (years)	73 [63–77]	74 [71–79]
Sex (female/male)	3/10	2/4
Height (cm)	172 [168–180]	179 [164–186]
Weight (kg)	80 [71–86]	82 [68–94]
Systolic BP (mmHg)	135 [110–143]	137 [126–152]
Diastolic BP (mmHg)	80 [70–83]	78 [66–80]
Smoker		
Active smoker or quit the past month	2 (15%)	1 (17%)
Have quit > 1 month ago	7 (54%)	3 (50%)
Never smoked	4 (31%)	2 (33%)
NYHA-class	I: 0 (0%)	I: 0 (0%)
	II: 5 (38%)	II: 3 (50%)
	III: 5 (38%)	III: 2 (33%)
	IV: 3 (23%)	IV: 1 (17%)
Perfusion gradients (%-counts/cm)	.13 [− 3.26–2.12]	− .36 [− 5.49–.22]
LVESV (echocardiography) (ml)	156 [131–191]	150 [109–196]
EF (echocardiography) (%)	22 [17–29]	23 [17–32]
Etiology		
Ischemic cardiomyopathy	7 (54%)	3 (50%)
Dilated cardiomyopathy	6 (46%)	3 (50%)
Nt-proBNP	1674 [386–4307]	1191 [419–3902]
Diabetes	0	0
Hypertension	6 (46%)	4 (67%)
Renal failure	3 (23%)	2 (33%)
Pharmacological treatment		
ACEI	10 (77%)	2 (33%)
ARB	3 (23%)	5 (83%)
Beta blocker	77 (84%)	6 (100%)
Aldosterone antagonist (Spironolactone)	5 (38%)	4 (67%)
Amidorane	1 (8%)	0
Digoxin Digitalis	1 (8%)	0
Statines	8 (62%)	4 (67%)
Warfarin	3 (23%)	1 (17%)
Anteroseptal-to-posterior delay	145 [50–195]*	101 [53–128]
Dyssynchrony	7 (%)	1 (%)
QRS duration		
< 150 ms	2 (15%)	1 (17%)
≥ 150 ms	11 (85%)	5 (83%)

Baseline data are presented as median [inter-quartile range] and percentage

*BP*, blood pressure; *NYHA*, New York Heart Association; *LV*, left ventricular; *ESV*, end-systolic volume; *EF*, ejection fraction; *Nt-proBNP*, N-terminal pro-B type natriuretic peptide; *ACEI*, angiotensin converting enzyme inhibitor; *ARB*, angiotensin receptor blockers

^*^Available on 12 patients

### Response and Non-response to CRT According to V/P SPECT and Echocardiography

#### V/P SPECT

**Figure 1:**
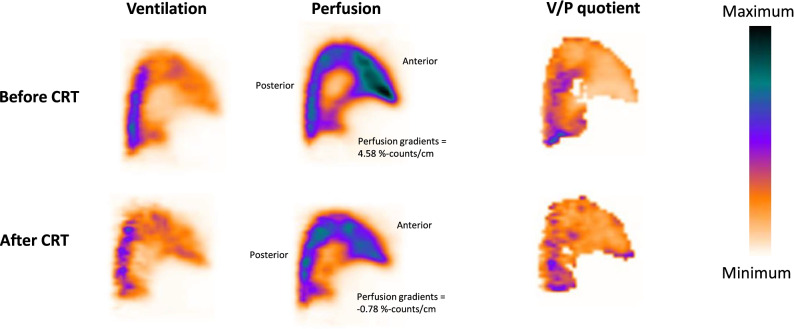
Ventilation/perfusion single-photon emission computed tomography (V/P SPECT) images (sagittal slices of the left lung) with the ventilation, perfusion, V/P quotient images from a patient before and after receiving CRT. The color scale to the right presents radioactive isotope distribution from minimum to maximum. Note that before CRT, the radioactive isotope uptake is highest in the anterior parts of the lungs, which results in a positive perfusion gradient. After CRT, the pulmonary perfusion pattern is normalized, and the perfusion gradient becomes negative. The ventilation images are not affected to the same degree

**Figure 2:**
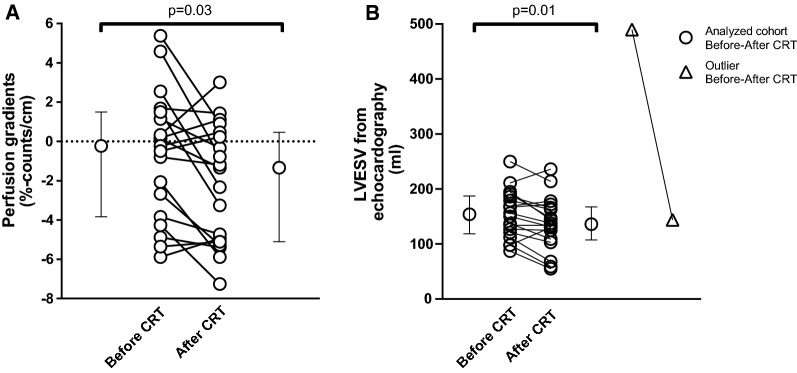
Perfusion gradients from ventilation/perfusion single-photon emission computed tomography (V/P SPECT) (A) and left-ventricular end-systolic volume (LVESV) from echocardiography (B) before and after cardiac resynchronization therapy (CRT) in patients with heart failure. The error bars represent median ± inter-quartile range. The dashed line in A represents the threshold value of perfusion gradients (0% counts/cm). The white triangle in B represents an outlier. This patient had very high LVESV by echocardiography. This patient was excluded from the statistical analysis; *P* value without the outlier = .01, *P* value with the outlier = .01

Figure 1 presents a V/P SPECT image of one of the patients before and after receiving CRT. There was a significant decrease in perfusion gradients after CRT in patients with heart failure (before: − .23 [− 3.84-1.5] %-counts/cm, after: − 1.34 [− 5.11-.46] %-counts/cm, *P* = .03, Figure 2A).

Sixty-eight percent of the patients (N = 13/19) showed improved perfusion gradients after receiving CRT (Table 2). There was an improvement in NYHA classification in 13/19 (68%) patients after receiving CRT. In five patients (26%), however, no improvement in NYHA classification was seen, and in one patient, there was a worsening in symptoms after CRT. The NYHA classifications for the patients before and after receiving CRT are listed in Table 1.

**Table 2:** Results of perfusion gradients from V/P SPECT vs improvement in NYHA classification, Minnesota living with heart failure (MLWHF) quality-of-life scoring system, LVESV, and LVEF (from echocardiography), as well as results of LVESV vs NYHA classification, MLWHF, LVEF, and perfusion gradients

	Perfusion gradients (improved)	Perfusion gradients (not improved)	*P* value	LVESV (improved)	LVESV (not improved)	*P* value
Improvement in NYHA (proportion of patients, N, %)	11/13 = 85%	2/6 = 33%	.0456	6/7 = 86%	7/12 = 58%	.3
Improvement in MLWHF (proportion of patients, N, %)	7/11 = 64% 2 patients missing	4/6 = 67%	1.0	5/7 = 71%	6/10 = 60% 2 patients missing	1.0
Improvement in LVEF (proportion of patients, N)	4/13 = 31%	1/6 = 17%	1.0	3/7 = 43%	2/12 = 17%	.3
Improvement in LVESV (proportion of patients, N, %)	5/13 = 38%	2/6 = 33%	1.0	–	–	–
Improvement in perfusion gradients (proportion of patients, N, %)	–	–	–	5/7 = 71%	8/12 = 67%	1.0

The majority of patients (85%; *n* = 11/13) who showed improvement in perfusion gradients after CRT also demonstrated an improvement in NYHA classification (functional capacity), *P* = .0456, Table 2. Change in perfusion gradients was not associated with changes in MLWHF (*P* = 1.0), LVEF (*P* = 1.0), or LVESV (*P* = 1.0), as presented in Table 2.

#### Echocardiography

**Figure 3:**
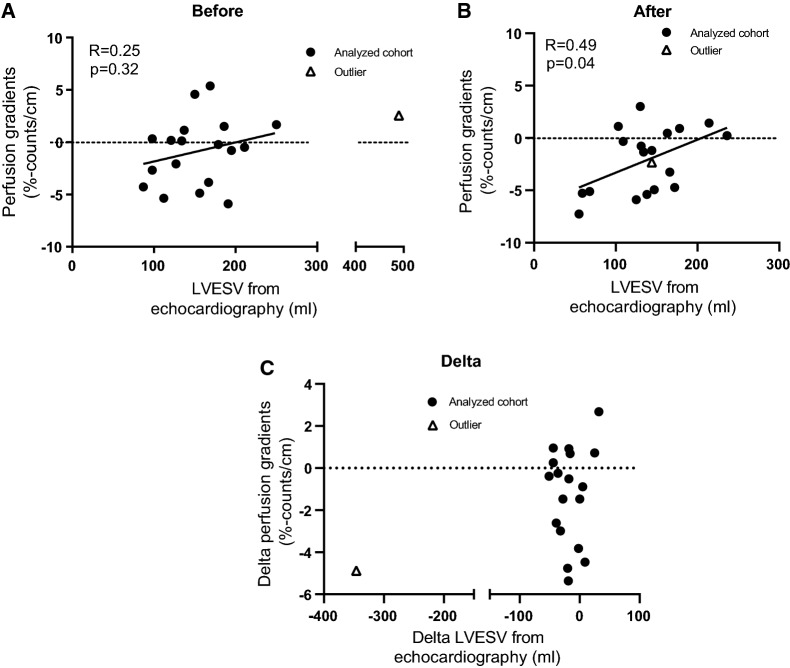
Perfusion gradients from ventilation/perfusion single-photon emission computed tomography (V/P SPECT) vs left-ventricular end-systolic volume (LVESV) from echocardiography in patients with heart failure (A) before and (B) after receiving CRT. Panel C presents the delta values of the perfusion gradients and LVESV. The solid black lines represent the linear regression line, and the dashed lines represent the cut-off value for the perfusion gradients (0% counts/cm). The white triangles represent an outlier. (A) The outlier was excluded from the analysis; without the outlier: *R* = .25, *P* = .32, *Y* = .018 × *X* − 3.67; with the outlier: *R* = .33, *P* = .17, *Y* = .012 × *X* − 2.75. (B) The outlier was excluded from the analysis; without the outlier: *R* = .49, *P* = .04, *Y* = .032 × *X* − 6.46; with the outlier: *R* = .49, *P* = .03, *Y* = .031 × *X* − 6.47

Seven out of nineteen patients (37%) showed an improvement in LVESV from echocardiography after CRT (Table 2). There was a significant decrease in LVESV by echocardiography after CRT (139 [108-168] mL) compared with before CRT: (159 [119-192] mL, *n* = 18, *P* = .01; Figure 2B). There was no association between improved and non-improved LVESV in relation to improvement in NYHA classification (*P* = .3), MLWHF (*P* = 1.0), LVEF (*P* = .3), and perfusion gradients (*P* = 1.0) (Table 2). There was no significant correlation between perfusion gradients and LVESV from echocardiography in patients before receiving CRT (*P* = .32, Figure 3A). A positive correlation was found between perfusion gradients and LVESV from echocardiography for patients after receiving CRT (*R* = .49 *P* = .04, Figure 3B). Five out of nineteen patients (26%) showed an improvement in LVEF from echocardiography after CRT.

#### NYHA classification versus other measures

There was neither an association between NYHA classification and 6-minute walk test (*P* = .07), LVEDV (*P* = .80) as well as LVEF (*P* = 1.0) before receiving CRT, nor after the treatment; 6-minute walk test (*P* = .16), LVEDV (*P* = .14) and LVEF (*P* = .18). The 6-minute walk test was only available in 17 patients before and 15 patients after receiving CRT.

## Discussion

This is the first study to use perfusion gradients from V/P SPECT to evaluate patients with heart failure before and after CRT. The main findings of this study were (1) perfusion gradients from V/P SPECT improved after CRT; (2) improved perfusion gradients after CRT were associated with improvements in functional capacity according to NYHA classification; and 3) more patients showed improvement in perfusion gradients from V/P SPECT after CRT compared with LVESV on echocardiography.

### Improved Perfusion Gradients After CRT

In the current study, CRT led to improved perfusion gradients from V/P SPECT in the majority of patients (68%; *n* = 13/19). This is in alignment with a recently-published study where perfusion gradients from V/P SPECT showed that the deranged pulmonary perfusion pattern seen in severe heart failure was normalized after heart transplantation using PAWP as the reference method.^14^ In a study from 2008, V/P SPECT showed that anti-congestive treatment led to an improved and normalized pulmonary perfusion pattern in patients with heart failure.^18^ The pulmonary perfusion pattern is known to be altered in patients with left heart failure, most likely because they develop pulmonary interstitial edema due to backward failure. This obstructs the capillary blood flow which leads to redistribution of pulmonary perfusion from posterior to anterior parts of the lungs in patients lying in a supine position.^13,18^ This phenomenon can be assessed and quantified using V/P SPECT.^13,18^

All patients in the present study received optimal pharmacological treatment. Despite this, only 10 of the 19 patients (53%) had normal (negative) perfusion gradients before CRT. Six of the ten patients (60%) with normal perfusion gradients were further normalized/improved after CRT, two worsened but were still normal (negative perfusion gradients), and the remaining two worsened in perfusion gradients after treatment to a level indicating newly established pulmonary congestion. According to the present study, perfusion gradients from V/P SPECT provide additional information that could help the understanding of the physiological effects of CRT in individual patients. This finding highlights the potential advantage of the perfusion gradients from V/P SPECT as a user-independent, non-invasive, and quantitative measure that can be regarded as a surrogate of invasive PAWP.^13,14^ Even though the majority of patients showed good response to CRT regarding perfusion gradients, it is yet to be shown if improved perfusion gradients are a predictor of decreased risk for hospitalization and long-term clinical outcome. This can be validated in a larger cohort in future studies. Future studies would also be useful to identify patients who will not respond to treatment before receiving CRT and establish if perfusion gradients could complement the currently used diagnostic methods in the follow-up after CRT.

### Perfusion Gradients and Improvement in NYHA Classification

An improvement in perfusion gradients was associated with an improvement in functional NYHA classification in most of the patients (85%). As the perfusion gradients were normal before treatment in five of these patients but showed further improvement after CRT, perfusion gradients alone could not be used to predict response to CRT. Perfusion gradients can, however, be used in the follow-up after CRT to objectively assess if the pulmonary perfusion pattern and signs of pulmonary congestion improves. V/P SPECT has shown to be more accurate than chest x-ray to diagnose pulmonary congestion.^13^

The current study is a sub-study to the CRT clinic trial.^15^ The proportion of patients who showed improvement in LVESV after CRT was only 37% (7/19) in the current study which is a smaller proportion than the main study (56%).^15^ LVESV was not associated with an improvement in NYHA classification after CRT. A lower proportion of patients showed improvements in LVESV by echocardiography after CRT in the current sub-study compared with perfusion gradients (37% vs 68%, respectively). An improvement in LVESV after CRT is correlated with improved long-term prognosis regarding future hospitalization events.^23^ The present study, as well as previous study, has shown a discrepancy between clinical and echocardiographic improvement (a reduction in LVESV), and a greater rate of clinical improvement was seen compared with LVESV reduction.^24^

### A Possible Rationale for the Use of Perfusion Gradients in the Follow-up of CRT

CRT mainly aims to treat the negative effects of dyssynchrony seen in patients with heart failure and LBBB. The short-term goal is to achieve a coordinated contraction of the ventricles and improve cardiac output. Over months, CRT also results in positive structural and functional changes, known as reverse remodeling, which further improves the heart function. Reverse remodeling results in decreased LV size, which can be seen with echocardiography as decreased LVESV. An improved LV function would also lead to a decrease in the degree of backward failure seen in heart failure. As the current study shows, this results in an improvement in lung perfusion pattern. Perfusion gradients from V/P SPECT evaluate the functional treatment effects on the heart and on pulmonary circulation. Therefore, the perfusion gradients could, at least in theory, be less dependent on actual macro-anatomical changes and more sensitive to the total functional capacity of the heart; however, this is yet to be shown.

According to the results of the current and the PROSPECT study,^24^ the use of echocardiography alone would indicate that a low number of patients benefit from CRT. As this is not in agreement with the positive effects seen especially on quality-of-life and morbidity,^2^ there is a need for complementary methods to evaluate CRT response.

### Limitation

As the current study is a sub-study to a published study where we added a V/P SPECT examination before and after CRT, the number of patients is small, and this is a limitation. The comparison between the surrogate measures, perfusion gradients, and LVESV, in our material is a limitation. However, perfusion gradients derived from V/P SPECT have previously been validated against invasive measurement of PAWP.^13,14^ It would have been valuable to validate the perfusion gradients against PAWP in the current study, and this could be considered to be done as a future study. Right heart catheterization is, however, an invasive examination. The number of LVESV responders in this sub-study was lower than in the main study (CRT clinic). The interpretation of the associations found, e.g., between improvement of NYHA classification and perfusion gradients on the one hand and a lack of association between NYHA classification and LVESV on the other hand, should, therefore, be viewed with caution, and the results should be confirmed in larger studies. Future studies are needed to investigate the perfusion gradients as an outcome measure regarding hospitalization and death.

## New Knowledge Gained

This is a novel study to non-invasively and objectively follow-up patients with heart failure after receiving CRT. The study shows that perfusion gradients from V/P SPECT can objectively quantify the improvement of patients with heart failure after receiving CRT and are better than echocardiography in demonstrating an improvement after the treatment.

## Conclusion

Perfusion gradients from V/P SPECT are a promising quantitative, user-independent method in the assessment of CRT response in patients with heart failure. The study demonstrated an improvement of perfusion gradients in a larger proportion of the patients after CRT compared with echocardiography. Moreover, improved perfusion gradients were associated with an improvement of patients’ functional capacity, according to NYHA. Perfusion gradients could have an added value to the currently used evaluation methods in the follow-up of patients with heart failure after receiving CRT.

## Supplementary Information

Below is the link to the electronic supplementary material.

Supplementary file 1 (PPTX 1085 kb)

Supplementary file 2 (MP3 1935 kb)

## Abbreviations

CRT: Cardiac resynchronization therapy
EF: Ejection fraction
LVESV: Left-ventricular end-systolic volume
MLWHF: Minnesota living with heart failure
NYHA classification: New York Heart Association Classification
PAWP: Pulmonary artery wedge pressure
V/P SPECT: Ventilation/perfusion single-photon emission computed tomography
