# Supplementary material for: Pulmonary perfusion and NYHA classification improve after cardiac resynchronization therapy
Source: J Nucl Cardiol. 2021 Nov 8;29(6):2974–83. doi: 10.1007/s12350-021-02848-8 (PMC9834347; doi:10.1007/s12350-021-02848-8)
Supplement: Supplementary file 1 — (PPTX 1085 kb) [file 12350_2021_2848_MOESM1_ESM.pptx]

## Slide 1
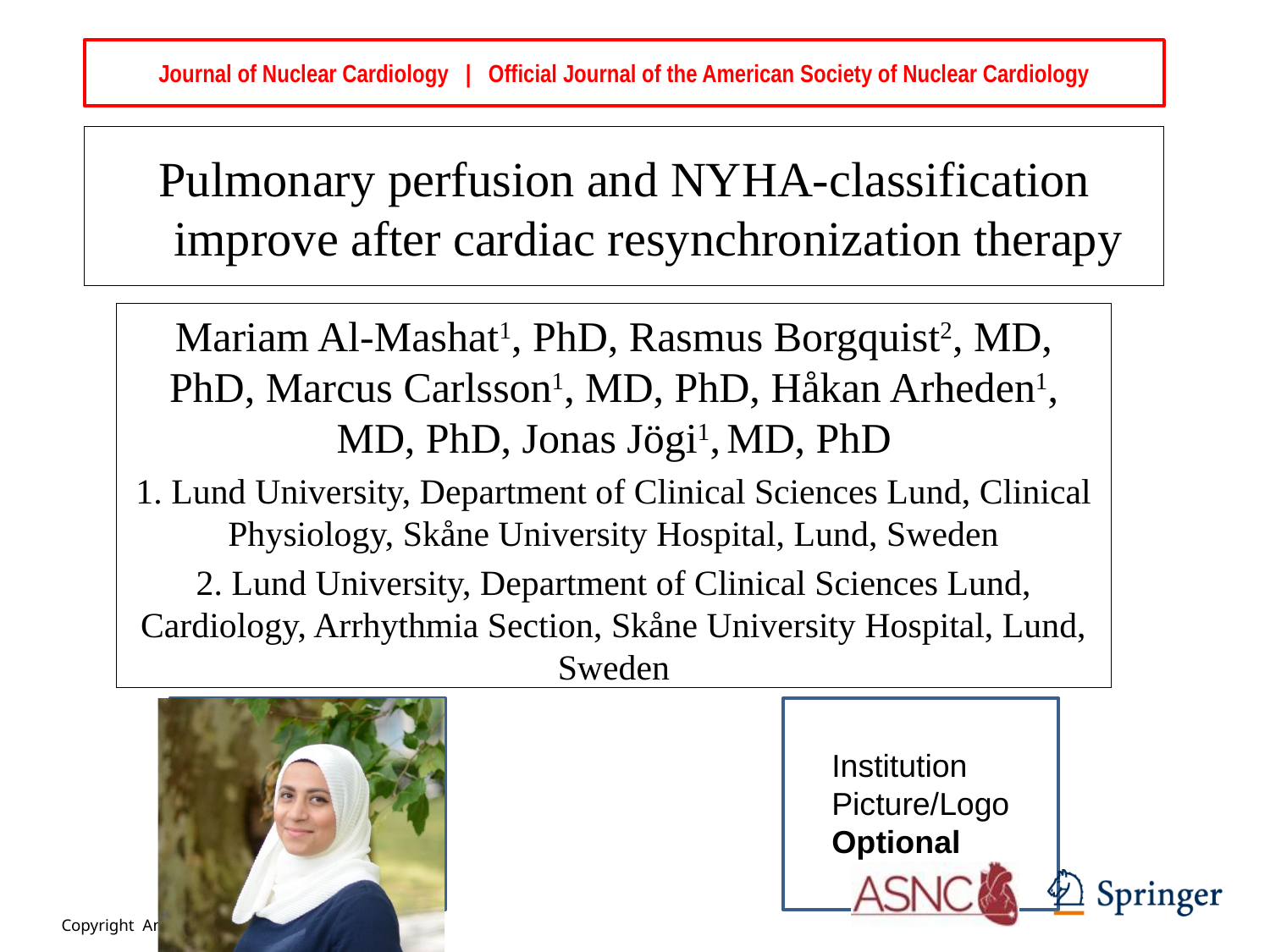

Journal of Nuclear Cardiology | Official Journal of the American Society of Nuclear Cardiology
# Pulmonary perfusion and NYHA-classification improve after cardiac resynchronization therapy
Mariam Al-Mashat1, PhD, Rasmus Borgquist2, MD, PhD, Marcus Carlsson1, MD, PhD, Håkan Arheden1, MD, PhD, Jonas Jögi1, MD, PhD
1. Lund University, Department of Clinical Sciences Lund, Clinical Physiology, Skåne University Hospital, Lund, Sweden
2. Lund University, Department of Clinical Sciences Lund, Cardiology, Arrhythmia Section, Skåne University Hospital, Lund, Sweden
Head shot of author
required
Institution
Picture/Logo
Optional
Copyright American Society of Nuclear Cardiology

## Slide 2
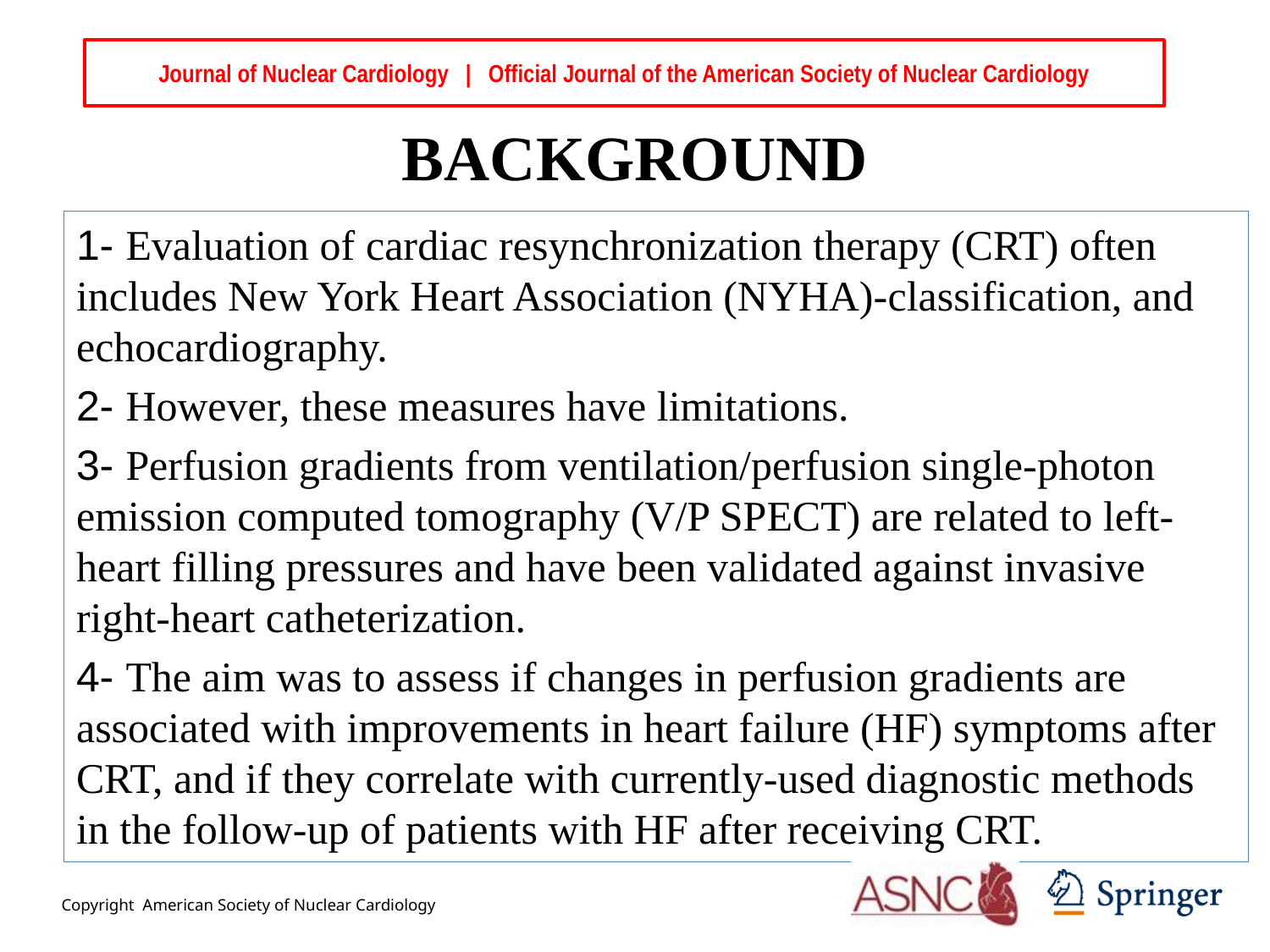

Journal of Nuclear Cardiology | Official Journal of the American Society of Nuclear Cardiology
# BACKGROUND
1- Evaluation of cardiac resynchronization therapy (CRT) often includes New York Heart Association (NYHA)-classification, and echocardiography.
2- However, these measures have limitations.
3- Perfusion gradients from ventilation/perfusion single-photon emission computed tomography (V/P SPECT) are related to left-heart filling pressures and have been validated against invasive right-heart catheterization.
4- The aim was to assess if changes in perfusion gradients are associated with improvements in heart failure (HF) symptoms after CRT, and if they correlate with currently-used diagnostic methods in the follow-up of patients with HF after receiving CRT.
Copyright American Society of Nuclear Cardiology

## Slide 3
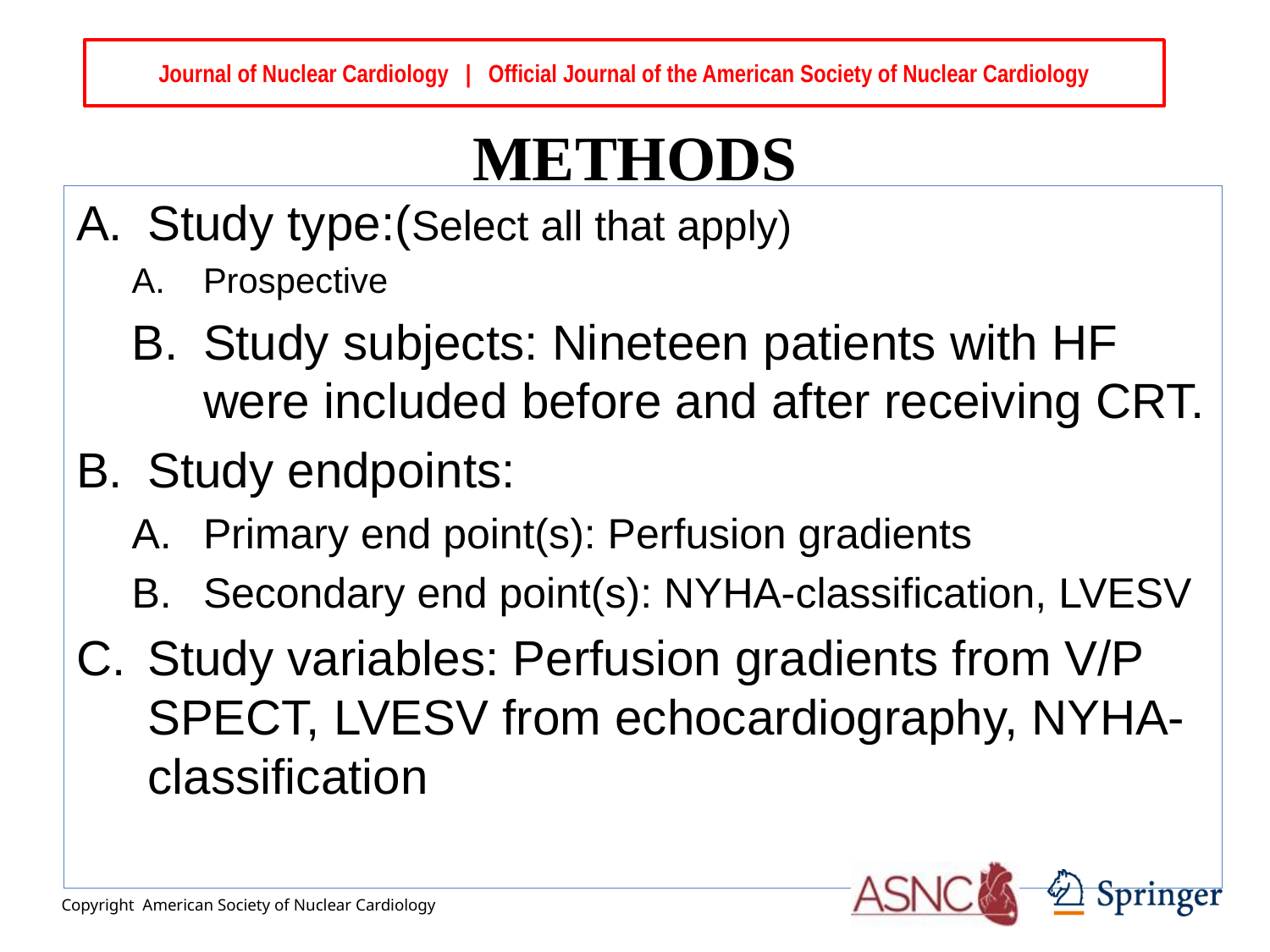

Journal of Nuclear Cardiology | Official Journal of the American Society of Nuclear Cardiology
# METHODS
Study type:(Select all that apply)
Prospective
Study subjects: Nineteen patients with HF were included before and after receiving CRT.
Study endpoints:
Primary end point(s): Perfusion gradients
Secondary end point(s): NYHA-classification, LVESV
Study variables: Perfusion gradients from V/P SPECT, LVESV from echocardiography, NYHA-classification
Copyright American Society of Nuclear Cardiology

## Slide 4
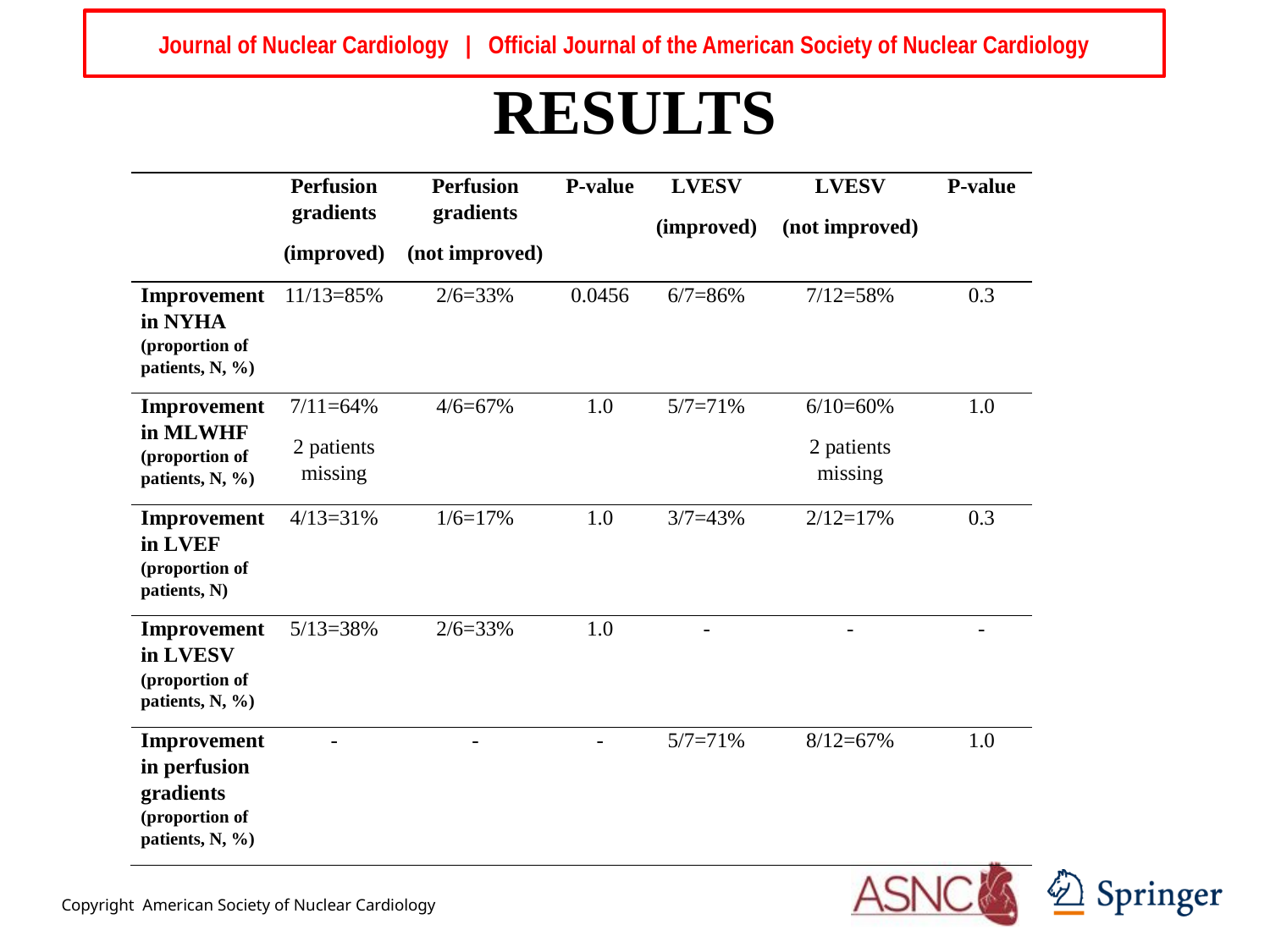

Journal of Nuclear Cardiology | Official Journal of the American Society of Nuclear Cardiology
# RESULTS
Copyright American Society of Nuclear Cardiology

## Slide 5
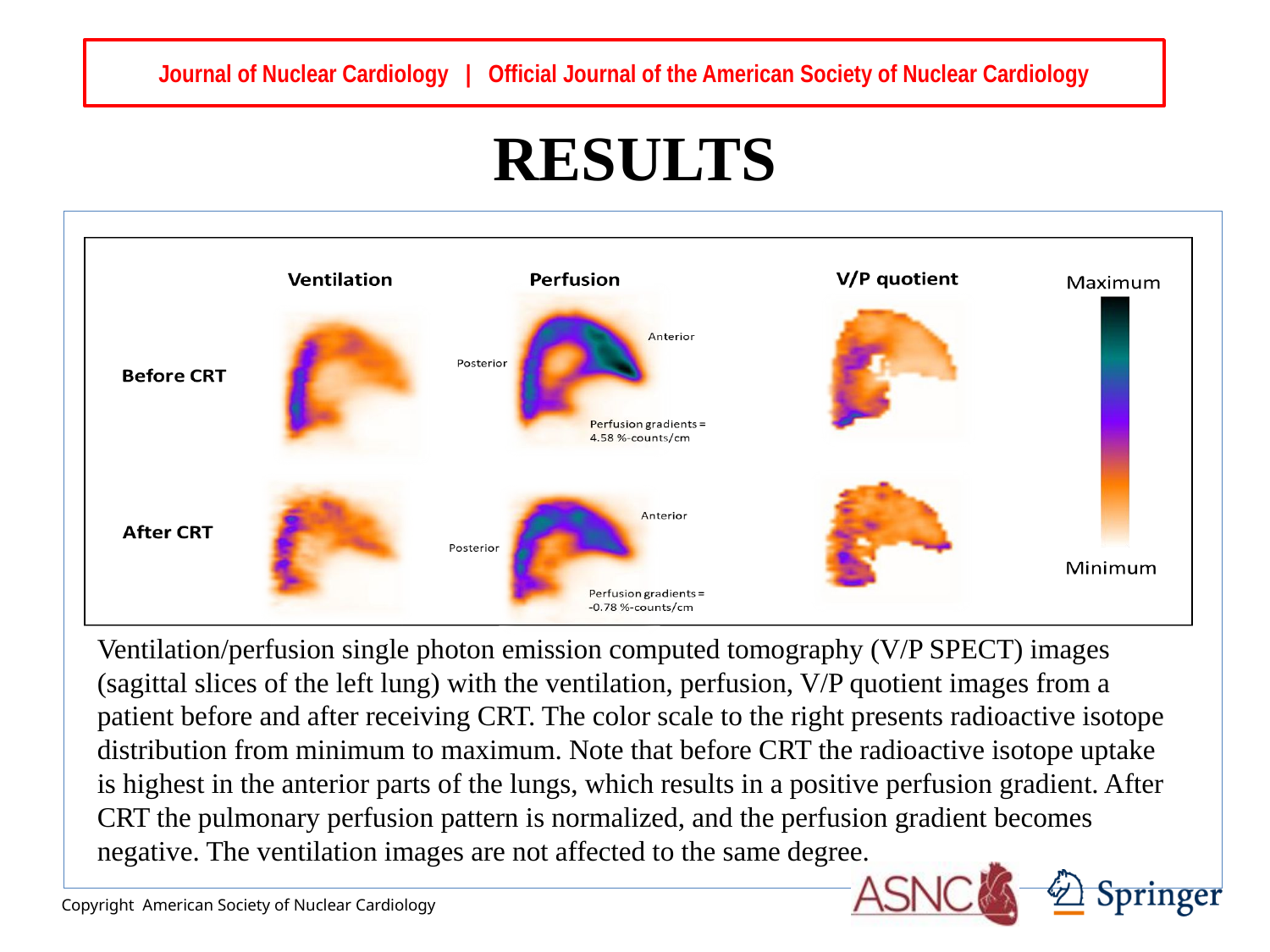

Journal of Nuclear Cardiology | Official Journal of the American Society of Nuclear Cardiology
# RESULTS
Ventilation/perfusion single photon emission computed tomography (V/P SPECT) images (sagittal slices of the left lung) with the ventilation, perfusion, V/P quotient images from a patient before and after receiving CRT. The color scale to the right presents radioactive isotope distribution from minimum to maximum. Note that before CRT the radioactive isotope uptake is highest in the anterior parts of the lungs, which results in a positive perfusion gradient. After CRT the pulmonary perfusion pattern is normalized, and the perfusion gradient becomes negative. The ventilation images are not affected to the same degree.
Copyright American Society of Nuclear Cardiology

## Slide 6
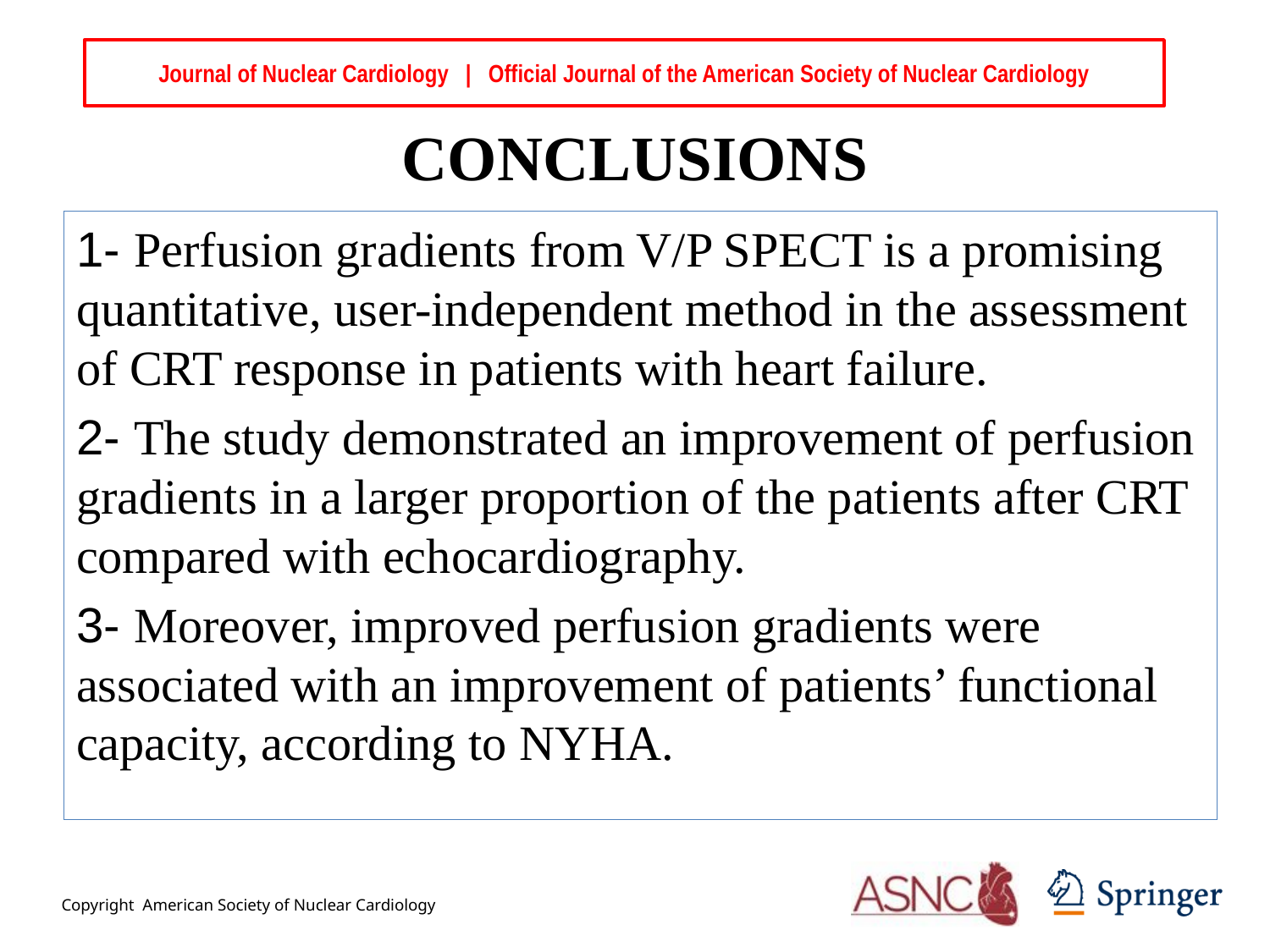

Journal of Nuclear Cardiology | Official Journal of the American Society of Nuclear Cardiology
# CONCLUSIONS
1- Perfusion gradients from V/P SPECT is a promising quantitative, user-independent method in the assessment of CRT response in patients with heart failure.
2- The study demonstrated an improvement of perfusion gradients in a larger proportion of the patients after CRT compared with echocardiography.
3- Moreover, improved perfusion gradients were associated with an improvement of patients’ functional capacity, according to NYHA.
Copyright American Society of Nuclear Cardiology
